# Antibacterial and anticancer activities of acetone extracts from in vitro cultured lichen-forming fungi

**DOI:** 10.1186/s12906-017-1819-8

**Published:** 2017-06-07

**Authors:** Agnieszka Felczykowska, Alicja Pastuszak-Skrzypczak, Anna Pawlik, Krystyna Bogucka, Anna Herman-Antosiewicz, Beata Guzow-Krzemińska

**Affiliations:** 1 Department of Molecular Biology, University of Gdańsk, Wita Stwosza 59, 80-308 Gdańsk, Poland; 2 Present address: Chair and Department of Physiology, Medical University of Gdańsk, Dębinki 1, 80-211 Gdańsk, Poland; 3 Present address: Department of Medical Biology and Genetics, University of Gdańsk, Wita Stwosza 59, 80-308 Gdańsk, Poland; 4 Present address: Department of Bacterial Molecular Genetics, University of Gdańsk, Wita Stwosza 59, 80-308 Gdańsk, Poland; 5 Present address: Department of Plant Taxonomy and Nature Conservation, University of Gdańsk, Wita Stwosza 59, 80-308 Gdańsk, Poland

**Keywords:** Antibacterial activity, Antiproliferative effect, Apoptosis, Lichen, MIC, MBC

## Abstract

**Background:**

Lichens that were used in traditional medicine for ages produce numerous secondary metabolites, however our knowledge about biological activities of substances secreted by separated bionts is scarce. The main objectives of this study were to isolate and find optimal conditions for the growth of mycelia from three common lichen-forming fungi, i.e. *Caloplaca pusilla, Protoparmeliopsis muralis* and *Xanthoria parietina* and to evaluate antibacterial and antiproliferative activities of their acetone extracts.

**Methods:**

Agar disc diffusion and broth microdilution methods were used to test antimicrobial activity against six species of bacteria. MTT method, flow cytometry assay and DAPI staining were applied to test antiproliferative activity of selected extracts against MCF-7 (human breast adenocarcinoma), PC-3 (human prostate cancer) and HeLa (human cervix adenocarcinoma) cancer cells.

**Results:**

*P. muralis* strongly inhibited the growth of Gram-positive bacteria, i.e. *Bacillus subtilis*, *Enterococcus faecalis, Staphylococcus aureus* and *Staphylococcus epidermidis* (MICs from 6.67 to 100.00 μg mL^−1^). *X. parietina* grown on PDA and G-LBM media decreased HeLa or MCF-7 cancer cells viability with IC_50_ values of about 8 μg mL^−1^, while *C. pusilla* grown on G-LBM medium showed the highest potency in decreasing MCF-7 (7.29 μg mL^−1^), PC-3 (7.96 μg mL^−1^) and HeLa (6.57 μg mL^−1^) cancer cells viability. We also showed induction of apoptosis in HeLa, PC-3 and MCF-7 cell lines treated with increasing concentrations of *C. pusilla* extract.

**Conclusion:**

We showed that selected acetone extracts demonstrated a strong antimicrobial and anticancer effects that suggests that aposymbiotically cultured lichen-forming fungi can be a source of antibacterial and antiproliferative compounds.

## Background

Lichens are the symbiotic phenotype of nutritionally specialized fungi (mycobiont) that derive fixed carbon from green algae and/or cyanobacteria (photobionts) and are present in ecosystems worldwide [1]. In nature they form thalli composed of at least two completely different organisms. Lichens are known to produce numerous secondary metabolites and developments in analytical techniques have allowed the identification of over 1000 lichen substances [2]. Among them one of the most widely studied is usnic acid which exhibits a wide range of biological properties, e.g. antibacterial, antifungal, antimitotic activities [3, 4].

Lichens provide food especially for animals but also for humans. They are used in the production of perfumes, alcohol and dyes. Numerous lichens have been used in folk medicines by cultures across the world, particularly in temperate and arctic regions (for review see Crawford [5]). Since many lichens exhibit antibiotic, antitumor, antimutagenic, antifungal, antiviral, enzyme inhibitory and plant growth inhibitory properties [6] they can be a potential source of chemicals useful in pharmaceutical industry or agriculture [7–11].

Many difficulties were encountered in sourcing substantial quantities of lichen substances required for various applications. As lichens are slow-growing organisms their intensive harvesting is not ecologically acceptable because lichen populations recover extremely slowly [12]. Moreover, many species are endangered due to climate change and air pollution, therefore included in Red Lists. However, numerous mycobionts and their photobionts can be grown in cultures aposymbiotically or in a symbiotic state [13–15], but for many lichen-forming fungi culture conditions remain unknown [15].

Although the properties of secondary metabolites from lichens are continuously studied [11, 16, 17] our knowledge about biological activities of substances secreted by separated bionts is scarce. Moreover, utilisation of secondary compounds directly from the lichen is neither feasible nor sustainable due to slow growth of many lichens [12]. Therefore, it would be of interest to grow them under laboratory conditions to prevent their over-exploitation for industrial purposes, especially if they would produce a wide range of useful substances.

Cancer is a major cause of death worldwide and the efforts to develop effective treatment continue using both synthetic and naturally occurring compounds. Moreover, due to resistance of numerous bacteria to commonly used antibiotics new antibacterial agents are continuously searched, among them bioactive compounds from plants or other organisms, such as lichens.

The major objectives of this study were: 1) to obtain mycelial cultures of three common lichen-forming fungi, i.e. *Caloplaca pusilla* Zahlbr.*, Protoparmeliopsis muralis* (Schreb.) Choisy and *Xanthoria parietina* (L.) Th.Fr.; 2) to test the impact of culture conditions on the growth of mycelia; 3) to evaluate antibacterial activity of mycelial extracts against several Gram-positive or Gram-negative bacterial strains and determine MIC and MBC values; 4) to test antiproliferative activity of selected extracts toward (i) MCF-7 human breast cancer cells, (ii) HeLa cervical cancer cells and (iii) PC-3 prostate cancer cells.

## Methods

### Lichen material

Samples of lichens *Protoparmeliopsis muralis, Caloplaca pusilla* and *Xanthoria parietina* were collected in Poland and were identified by Beata Guzow-Krzemińska. They are preserved in the Department of Plant Taxonomy and Nature Conservation of the University of Gdańsk.

### Mycobiont isolation

Mycobionts were isolated from spores according to Ahmadjian [18] method with an additional pre-washing step of the apothecia in water with a small drop of Tween 80 detergent [19]. The germinating spores were transfered to nutrient media, i.e. G-LBM [20], Malt extract-Yeast extract medium [18] and Potato-Dextrose Agar (PDA, Merck). The cultures were kept in the culture chamber under the following cycle: 20 °C/14 h and 10 °C/10 h and were regularly checked for visible contaminants. Well-developed mycelia were subsequently used for sub-culturing. The mycelia were gently homogenized with water and the suspension was transferred with a Pasteur pipette to a new Petri dish containing one of the following nutrient media: G-LBM [20], modified G-LBM with a higher concentration of biotine 80 μg L^−1^ (G-LBM+), Potato-Dextrose Agar (PDA) and malt extract-yeast extract medium (MY) [18] The cultures were kept in the culture chamber under the following cycle: 20 °C/14 h and 10 °C/10 h for 2 months and then were used for further experiments.

### DNA analysis

DNA extracts were used for PCR-amplification of ITS rDNA marker using ITS1F [21] and ITS4 primers [22]. 25 μL of PCR mix contained 1 U of Taq polymerase (Thermo Scientific), 0.2 mM of each of the four dNTP’s, 0.5 μM of each primer and 10–50 ng of genomic DNA. PCR amplifications were performed using a Mastercycler (Eppendorf) with the following program: initial denaturation at 95 °C for 5 min followed by 35 cycles at 95 °C for 40 s, 54 °C for 45 s and 72 °C for 1 min, and a final elongation step at 72 °C for 10 min. PCR products were visualized on agarose gels in order to determine DNA fragment lengths. Subsequently, 5 μL of PCR products were treated with 10 units of Exonuclease I and 1 unit of FastAP™ Thermosensitive Alkaline Phosphatase enzymes (Thermo Scientific) to degrade primers and dephosphorylate dNTPs. Treatment was carried out for 15 min at 37 °C, followed by a 15 min incubation at 85 °C to completely inactivate both enzymes. Sequencing of each PCR product was performed using Macrogen sequencing service (http://www.macrogen.com). For sequencing ITS1F primer [21] was used.

The newly determined ITS rDNA sequences from mycelia of *Caloplaca pusilla*, *Protoparmeliopsis muralis* and *Xanthoria parietina* were compared to the sequences available in GenBank using BLASTn search [23] in order to confirm the identity of the mycobiont.

### Preparation of the acetone extracts

Mycelia were cut out of the medium, weighted and air dried at 40 °C. Dry pieces of the mycelia of the investigated lichens were pulverized using mortar and weighted again. Equivalent amount of the acetone (POCh, Poland) was added to each sample. The extraction was performed using ultrasonic water bath for 30 min and then the solutions were incubated at room temperature overnight. The extracts were filtered using Whatman filter paper (No. 1) and then the acetone was evaporated from the filtrate to dryness. The dry mass was weighted and dissolved in DMSO (Sigma-Aldrich) to the concentration of 10 mg mL^−1^ and sterilized by filtration through 0.45 μm Millipore filters.

### Bacterial and fungal strains

The following bacterial strains were used for antibacterial activity test: *Bacillus subtilis* wt 168, *Escherichia coli* 32,241/09, *Enterococcus faecalis* 773, *Klebsiella pneumoniae* ESBL(+)14,872/09 (AM-resistant, SXT-resistant, CZ-resistant, CTX-resistant, CXM-resistant, NF-resistant), *Pseudomonas aeruginosa* 6121/10, *Staphylococcus aureus* 6285/10, *Staphylococcus epidermidis* ATCC 14990.

These strains were obtained from the collection maintained in the Department of Molecular Biology of the University of Gdańsk. Bacteria were cultured in LB nutrient medium at 37 °C.

### Antimicrobial assays

#### Agar disc diffusion method

The antimicrobial activity was evaluated using agar disc diffusion method. The bacteria were inoculated in LB medium and incubated overnight. Bacteria were sub-cultured in LB liquid medium at 37 °C to OD_600_ = 0.2. Then 100 μL of inoculum was spread on a Petri dish. Three μL of the extract was applied to a sterilized Whatman filter paper disc placed on LB agar plate with appropriate bacterial strain. Netilmycine 30 was used as a positive control for all bacteria tested. The plates were incubated at 37 °C for 16–18 h. The diameter of the zones of inhibition around each disc was measured. The experiments were performed at least thrice and mean diameter of the zone of inhibition was taken as a measure of the antibacterial activity.

#### Broth microdilution method

Quantitative antibacterial activities were determined by measuring minimal inhibitory concentration (MIC) and minimal bactericidal concentration (MBC) using broth microdilution method in 96-well microtiter plates. The inoculum of the bacterial strains was prepared from overnight cultures and bacterial suspensions were adjusted to 0.5 McFarland turbidity standard. The inoculum was then diluted 100 times. A series of dilutions of extracts with concentrations ranging from 0 to 100 μg mL^−1^ were added to each well and 100 μL of inoculum was added and the wells were filled up with appropriate amount of LB medium. The final volume in each well was 200 μL. DMSO and usnic acid (Sigma-Aldrich) were used as controls. The plates were incubated at 37 °C for 16–18 h. The MIC value of each sample was determined. The MBC value was determined by sub-culturing samples from the wells with concentrations above the MIC on new plates with LB agar medium. The experiments were performed at least thrice.

### Reagents for analysis of antiproliferative effects

Tissue culture media, fetal bovine serum, DMSO, penicillin/streptomycin antibiotic mixture, acridine orange, ethidium bromide, thiazolyl blue tetrazolium bromide (MTT) were from Sigma (St. Louis, MO).

### Cell culture conditions

Human breast adenocarcinoma cell line MCF-7 was from CLS Cell Lines Service GmbH (Eppelheim, Germany). Human cervix adenocarcinoma cell line HeLa was obtained from Department of Biochemistry, Institute for Cancer Research, The Norwegian Radium Hospital, Oslo. Monolayer cultures of MCF-7 and HeLa cells were maintained in RPMI 1640 medium supplemented with 10% (*v*/v) fetal bovine serum and antibiotics. PC-3 cells were maintained in F12-K medium supplemented with 10% (*v*/v) fetal bovine serum and antibiotics. Each cell line was maintained at 37 °C in a humidified atmosphere with 5% CO2.

### Cell viability assay

Cell viability was determined by MTT method. Cells were seeded at a density of 2 × 10^3^ per well of 96-well plate and allowed to attach overnight. The medium was replaced with fresh medium supplemented with 1, 2, 4, 6, 8, 10 μg mL^−1^ of lichen extracts for 48 h. Before the end of treatment, 25 μL of MTT solution (4 mg mL^−1^) was added to each well. After 3 h of incubation, medium was removed and formazan crystals were dissolved in 100 μL of DMSO. Absorbance was measured at 570 nm (with reference wavelength 660 nm) in Victor^3^ microplate reader. The results are shown as the mean ± SE from three independent experiments performed in triplicates.

### Cell death assay

The fraction of apoptotic and necrotic cells was evaluated using flow cytometric detection of annexin V and 7-AAD positive cells as described previously [24]. 5 × 10^4^ cells were seeded in 6-well plates. After 24 h, cells were exposed to 0.1, 0.2, 0.5, 1, 2, 4, 6, 8, 10 μg mL^−1^ of lichen extracts for 48 h. Then, both medium and trypsynized cells were collected, centrifuged for 10 min, 400 x g. Percentage of cells in early (Annexin-V+/7-AAD-), late stages of apoptosis (Annexin-V+/7-AAD+) or necrotic cells (Annexin-V−/7-AAD+) was determined using Muse™ Cell Analyzer. The results are shown as the mean ± SE from three independent experiments.

### Morphological detection of apoptosis by DAPI staining

5 × 10^4^ cells were seeded on coverslips in 6-wells plates and incubated overnight. After 24 h, cells were treated with increasing concentrations of the analyzed extract for 48 h. The control cells were treated with the vehicle (DMSO)*.* After incubation cells were harvested, washed with PBS and fixed with 2% paraformaldehyde in PBS for 10 min. The cells were then stained with DAPI (1 μg/ml) for 2 min. The stained cells were spotted onto a slide. Next, glass coverslips were mounted onto glass microscope slides using mounting media and observed under a Leica DMI4000B fluorescence microscope.

### Statistical analysis

All data are shown as means ± standard error (SE) or ± standard deviation (SD) of at least three independent experiments. IC_50_ values were determined using linear regression in Microsoft Excel 2010 based on three independent experiments, each in duplicate. Significance of differences between control and treated cells in viability tests and cell death assay were analyzed with ANOVA and Dunnett’s multiple comparison post-hoc test using Statistica 12 (Statsoft). Difference was considered significant at *p* < 0.05 and is marked with *.

## Results

### Mycelial cultures

*Caloplaca pusilla, Protoparmeliopsis muralis* and *Xanthoria parietina* turned out to be cultivable, however, some of the cultures became contaminated by fungi or bacteria and those samples were discarded. The germinating spores were observed under the microscope and after 4 weeks of incubation the germinating spores were transferred to the nutrient media, i.e. G-LBM [20], malt extract-yeast (MY) extract medium [18] or potato-dextrose agar (PDA, Merck).

After 2 months of incubation the growth of mycelia was checked and the mycobionts were subcultured on G-LBM [20], modified G-LBM with a higher concentration of biotine of 80 μg L^−1^ (G-LBM+), malt extract-yeast extract medium (MY) [18] or potato-dextrose agar (PDA). Moreover, the identities of the mycelial cultures were checked using ITS rDNA sequencing followed by BLASTn analysis [23]. Based on comparison of each sequence with sequences deposited in the NCBI database it was found that the cultures obtained were *Caloplaca pusilla* (GenBank Acc. No. KY379231)*, Protoparmeliopsis muralis* (GenBank Acc. No. KY379232) and *Xanthoria parietina* (GenBank Acc. No. KY379230) fungi.

**Fig. 1:**
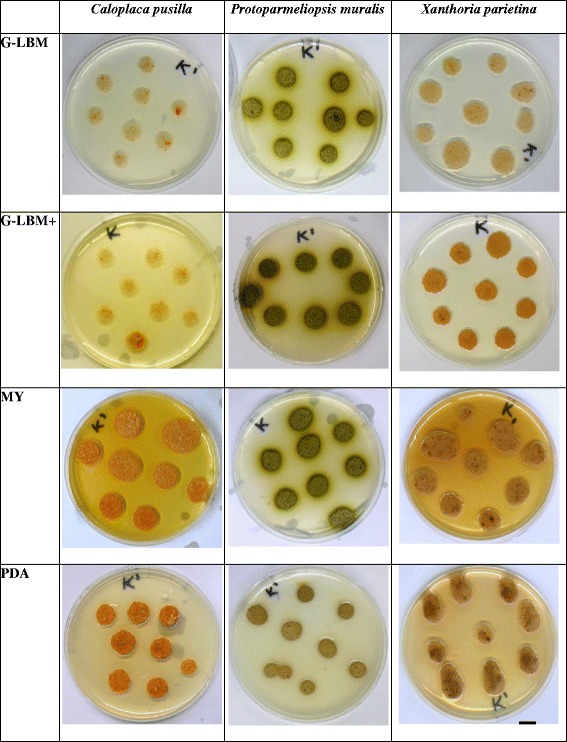
Effect of medium type on the growth of mycelia after 2 months of incubation. ^a^The mycelia of *Caloplaca pusilla, Protoparmeliopsis muralis* and *Xanthoria parietina* were cultured on G-LBM, modified G-LBM with a higher concentration of biotine of 80 μg L^−1^ (G-LBM+), Potato-Dextrose Agar (PDA) and malt extract-yeast extract medium (MY). Scale 1 cm

The growth of mycelia was checked every week and finally was documented after 2 months of incubation. Each fungus showed different growth rate and mycelium pigmentation (Fig. 1).

The growth of *Caloplaca pusilla* was very slow on both G-LBM and G-LBM+ media, while this mycobiont grew well on MY and PDA media, on which finally the mycelium became orange-pigmented with white crystals on the hyphae.

*Protoparmeliopsis muralis* was successfully cultured on all four media tested. *Protoparmeliopsis muralis* grew fast on G-LBM medium and the greenish pigmentation of the mycelium appeared after 6 weeks of subculturing. Finally, the mycelia became dark-green to almost brown and the pigment diffused to the medium around mycelial biomass (Fig. 1). Previous study showed that *P. muralis* can be successfully aposymbiotically grown on BBM with 1% glucose, G-LBM and Murashige-Scogg media, while its growth was inhibited on Saboraud 2% glucose medium [19].

In case of *Xanthoria parietina* the mycelial growth was observed on all four media tested but it was the most effective on MY and PDA medium. After 2 months of incubation the mycelia became yellow to light brown pigmented (Fig. 1).

### Antibacterial activities of acetone extracts

In this study we tested acetone extracts from in vitro cultured three species of lichen-forming fungi. The acetone is considered as a suitable solvent for extraction of phenolic compounds that might be produced by lichens, therefore we decided to make our analyses using acetone extracts. Their activities were qualitatively and quantitatively evaluated by the presence or the absence of inhibition zone and its diameter, MICs and MBCs values. We used an agar disc diffusion method to elucidate antibacterial activities of mycelial extracts and mean diameters of zones of inhibition from at least three replicates are given in Table 1.

**Table 1:** Mean diameter of zone of growth inhibition (mm) caused by each extract

	Medium^a^	*Bacillus subtilis*	*Staphylococcus aureus*	*Enterococcus faecalis*	*Staphylococcus epidermidis*	*Klebsiella pneumoniae*	*Escherichia coli*	*Pseudomonas aeruginosa*
*Protoparmeliopsis muralis*	PDA	6.00 ± 1.41^d^	n.d.	n.d.	n.d.	n.d.	n.d.	n.d.
	MY	15.33 ± 1.15	12.00 ± 1.00	5.67 ± 0.58	19.67 ± 1.53	n.d.	n.d.	n.d.
	G-LBM	15.50 ± 0.58	15.67 ± 0.58	9.25 ± 1.26	19.67 ± 0.58	n.d.	5.67 ± 1.15	5.67 ± 1.15
	G-LBM+	15.50 ± 0.58	16.67 ± 1.53	11.50 ± 1.91	21.33 ± 1.53	5.67 ± 1.15	6.33 ± 1.15	5.67 ± 1.15
	Pm thallus^b^	11.75 ± 0.5	8.00 ± 1.00	10.50 ± 0.58	15.67 ± 1.15	n.d.	5.67 ± 1.15	n.d.
*Xanthoria parietina*	PDA	n.d.	n.d.	n.d.	n.d.	n.d.	n.d.	n.d.
	MY	n.d.	n.d.	n.d.	5.67 ± 1.15	n.d.	n.d.	n.d.
	G-LBM	n.d.	n.d.	n.d.	5.67 ± 1.15	n.d.	n.d.	n.d.
	G-LBM+	n.d.	n.d.	n.d.	n.d.	n.d.	n.d.	n.d.
	Xp thallus^b^	n.d.	n.d.	n.d.	n.d.	n.d.	5.67 ± 1.15	n.d.
*Caloplaca pusilla*	PDA	5.25 ± 0.50	n.d.	n.d.	n.d.	n.d.	n.d.	n.d.
	MY	5.25 ± 0.50	n.d.	n.d.	n.d.	n.d.	5.67 ± 1.15	n.d.
	Cp thallus^b^	6.50 ± 1.00	n.d.	n.d.	n.d.	n.d.	5.67 ± 1.15	n.d.
	NET30^c^	23.33 ± 0.58	17.00 ± 1.00	14.75 ± 2.06	18 ± 1.00	17.67 ± 2.08	18.00 ± 1.00	23.00 ± 2.00

^a^The mycelia were cultured on G-LBM, modified G-LBM with a higher concentration of biotine of 80 μg L^−1^ (G-LBM+), Potato-Dextrose Agar (PDA) and malt extract-yeast extract medium (MY) [18]. Standard deviations are added

^b^Cp-*Caloplaca pusilla*, Pm- *Protoparmeliopsis muralis*, Xp-*Xanthoria parietina*

^c^The antibiotic Netilmycine 30 (NET30) was used as the control

^d^The experiments were performed at least in triplicate. n.d.-not determined

Extracts from *Caloplaca pusilla* and *Xanthoria parietina* did not show any antibacterial activity in agar disc diffusion tests. Extracts from *Protoparmeliopsis muralis* were the most active against Gram-positive bacterial strains i.e. *Bacillus subtilis* wt 168, *Enterococcus faecalis* 773, *Staphylococcus aureus* 6285/10 and *Staphylococcus epidermidis* ATCC 14990, while they were inactive against all tested Gram-negative bacteria (Table 1). Moreover, extract from mycelium grown on PDA medium did not show any antibacterial activity in agar disc diffusion test. Other extracts from *P. muralis* were the most active against *Staphylococcus epidermidis* (zone of inhibition ranging from 15.67 mm for extract from the lichen thallus to 21.33 mm for extract from mycelium grown on G-LBM+ medium) while they were the least active against *Enterococcus faecalis*. The activities of mycelial extracts from *P. muralis* were similar to those of antibiotic Netilmycine 30 (Table 1).

MICs and MBCs values were determined for each bacterium and it was found that extracts from *Caloplaca pusilla* and *Xanthoria parietina* did not show any antibacterial effect at the concentrations used. Moreover, *Protoparmeliopsis muralis* grown on PDA medium was inactive against all bacteria tested. In Table 2 mean MIC and MBC values determined for extracts from *Protoparmeliopsis muralis* and usnic acid that was used as a control are given. The extracts showed the inhibitory effect on the growth of Gram-positive bacteria, i.e. *Bacillus subtilis* wt 168 (MICs from 6.67 to 23.75 μg mL^−1^), *Enterococcus faecalis* 773 (MICs from 50 to 100 μg mL^−1^), *Staphylococcus aureus* 6285/10 (MICs from 21.70 to 26.70 μg mL^−1^) and *Staphylococcus epidermidis* ATCC 14990 (MICs from 6.67 to 25.00 μg mL^−1^). The minimal bactericidal concentrations were determined only for *Bacillus subtilis* (25 μg mL^−1^), *Staphylococcus aureus* (MBCs from 23.33 to 38.33 μg mL^−1^) and *Staphylococcus epidermidis* (MBCs from 25.00 to 35.00 μg mL^−1^) (Table 2).

**Table 2:** Minimal inhibitory concentration (MIC) and minimal bactericidal concentration (MBC) of extracts from *Protoparmeliopsis muralis*

Species	*Bacillus subtilis*		*Staphylococcus aureus*		*Enterococcus faecalis*		*Staphylococcus epidermidis*		*Escherichia coli*		*Klebsiella pneumoniae*		*Pseudomonas aeruginosa*	
Medium^a^	MIC	MBC	MIC	MBC	MIC	MBC	MIC	MBC	MIC	MBC	MIC	MBC	MIC	MBC
PDA	n.d.^b^	n.d.	n.d.	n.d.	n.d.	n.d.	n.d.	n.d.	n.d.	n.d.	n.d.	n.d.	n.d.	n.d.
MY	23.75	25.00	25.00	25.00	100.00	n.d.	25.00	25.00	n.d.	n.d.	n.d.	n.d.	n.d.	n.d.
G-LBM	21.25	25.00	26.70	38.33	50.00	n.d.	21.25	35.00	n.d.	n.d.	n.d.	n.d.	n.d.	n.d.
G-LBM+	6.67	25.00	21.70	23.33	50.00	n.d.	6.67	30.00	n.d.	n.d.	n.d.	n.d.	n.d.	n.d.
Usnic acid^c^	8.33	40.00	56.70	n.d.	100.00	n.d.	5.00	15.00	n.d.	n.d.	n.d.	n.d.	n.d.	n.d.

^a^The mycelia were cultured on G-LBM, modified G-LBM with a higher concentration of biotine of 80 μg L^−1^ (G-LBM+), Potato-Dextrose Agar (PDA) and malt extract-yeast extract medium (MY) [18]

^b^The experiments were performed at least in triplicate. n.d.-not determined

^c^Usnic acid was used as a control

### Screening of lichen-forming fungi extracts for potential anticancer activity

In this study 8 acetone lichen fungi extracts were examined for the anticancer potential toward (i) MCF-7 human breast cancer cell line, (ii) HeLa cervical cancer cell line and (iii) PC-3 prostate cancer cell line. Cells were exposed for 48 h to increasing concentrations of lichen extracts (from 1 to 10 μg mL^−1^) and the viability of the cancer cells was determined with the use of MTT test. As shown in Table 3, three extracts revealed anticancer activity with IC_50_ value lower than IC_50_ of usnic acid (6.57–8.41 vs 10.88–14.50 μg mL^−1^) i.e. extracts from *Xanthoria parietina* grown on PDA (labelled as Xp_PDA) and G-LBM (labelled as Xp_GLBM) media and *Caloplaca pusilla* grown on G-LBM medium (labelled as Cp_GLBM). Extracts from *Protoparmeliopsis muralis* did not show any anticancer activity in our study.

**Table 3:** Sensitivity (IC_50_) of different cancer cell lines to extracts after 48 h treatment

Extract name	IC_50_ [μg mL^−1^]		
	HeLa	MCF-7	PC-3
Xp_PDA^a^	8.41 ± 0.87	n.d.^c^	n.d.
Xp_GLBM	n.d.	8.14 ± 1.44	n.d.
Cp_PDA	n.d.	n.d.	n.d.
Cp_GLBM	6.57 ± 0.41	7.29 ± 1.04	7.96 ± 1.21
Usnic acid^b^	10.88 ± 2.28	12.64 ± 2.95	14.50 ± 2.71

^a^Extracts from mycelia of different lichen-forming fungi i.e. Cp-*Caloplaca pusilla,* Xp-*Xanthoria parietina* grown on different media (i.e. on G-LBM [20] and Potato-Dextrose Agar (PDA)

^b^Usnic acid was used as a control

^c^n.d.-not determined

**Fig. 2:**
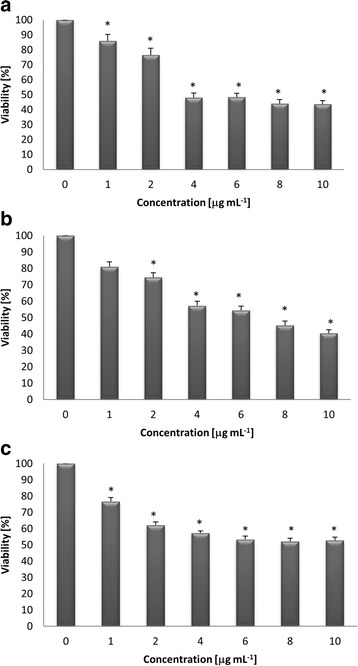
Effect of the extract on viability of HeLa (**a**), MCF-7 (**b**) and PC-3 (**c**) cancer cell lines. ^a^Extract from mycelium of *Caloplaca pusilla* grown on G-LBM medium was used. Cells were treated with different concentrations of the extract (1, 2, 4, 6, 8 and 10 μg mL^−1^) for 48 h. Each value is mean (±SE) of three experiments done in duplicate. Statistical significance determined with ANOVA and Dunnett’s post-hoc test is marked with *

Three out of 8 extracts showed antiproliferative effects on HeLa, MCF-7 and/or PC-3 cell lines (Table 3). Extract obtained from *Caloplaca pusilla* grown on GLBM medium revealed the highest potency in decreasing the cancer cell viability with IC_50_ values of 6.57 μg mL^−1^ for HeLa cell line, 7.29 μg mL^−1^ for MCF-7 and 7.96 μg mL^−1^ for PC-3 cell lines. The effect was dose-dependent (Fig. 2). As being the most active, the extract from mycelium of *Caloplaca pusilla* grown on G-LBM medium was chosen for further investigation.

### Extract from mycelium of *Caloplaca pusilla* grown on G-LBM medium induces the apoptosis in HeLa cells

**Fig. 3:**
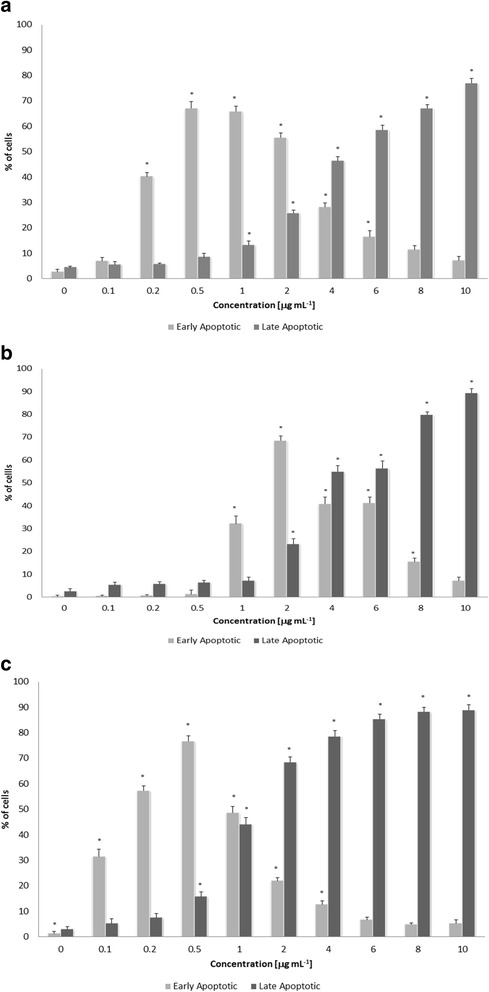
HeLa (**a**), MCF-7 (**b**) and PC-3 (**c**) cancer cells death assessed by labeling with annexin V and 7-AAD and flow cytometry. ^a^Cells were treated with different concentrations of extract from mycelium of *Caloplaca pusilla* grown on G-LBM medium (0, 0.1, 0.2, 0.5, 1, 2, 4, 6, 8 and 10 μg mL^−1^) for 48 h. Each value is mean (±SE) of three experiments done in triplicate. Statistical significance determined with ANOVA and Dunnett’s post-hoc test is marked with *

To verify whether a decrease in HeLa cell viability upon treatment with extract from *Caloplaca pusilla* grown on G-LBM medium is associated with an apoptosis induction, we examined the apoptotic and necrotic fraction using the flow cytometric detection of cells labeled with annexin V and 7-AAD. Annexin V marks apoptotic cells and 7-AAD dye stains cells with disintegrated membrane. Increasing apoptotic fractions (relative to control) were detected in all tested concentrations of the extract from mycelium of *Caloplaca pusilla* grown on G-LBM medium. The percentage of annexin V positive cells reached 70% in samples treated with 0.5–10 μg mL^−1^ extract (Fig. 3a). The HeLa cells exposed for 48 h to 0.2–2 μg mL^−1^ concentrations of extract from mycelium of *Caloplaca pusilla* grown on G-LBM medium were characterized by an increasing percentage of cells in early stage of apoptosis (Annexin-V+/7-AAD-). However, higher concentrations of the mycelial extract (4–10 μg mL^−1^) elevated amount of cancer cells in the late stages of apoptosis (Annexin-V+/7-AAD+). Similar trends were observed in PC-3 and MCF-7 cells, except that MCF-7 cells were more sensitive (significant fraction of apoptotic cells was detected already upon 0.1 μg mL^−1^ concentrations of the extract) while PC-3 cells were more resistant than HeLa cells (significant fraction of apoptotic cells was detected upon 1 μg mL^−1^ concentrations of the extract) (Fig. 3b and c).

**Fig. 4:**
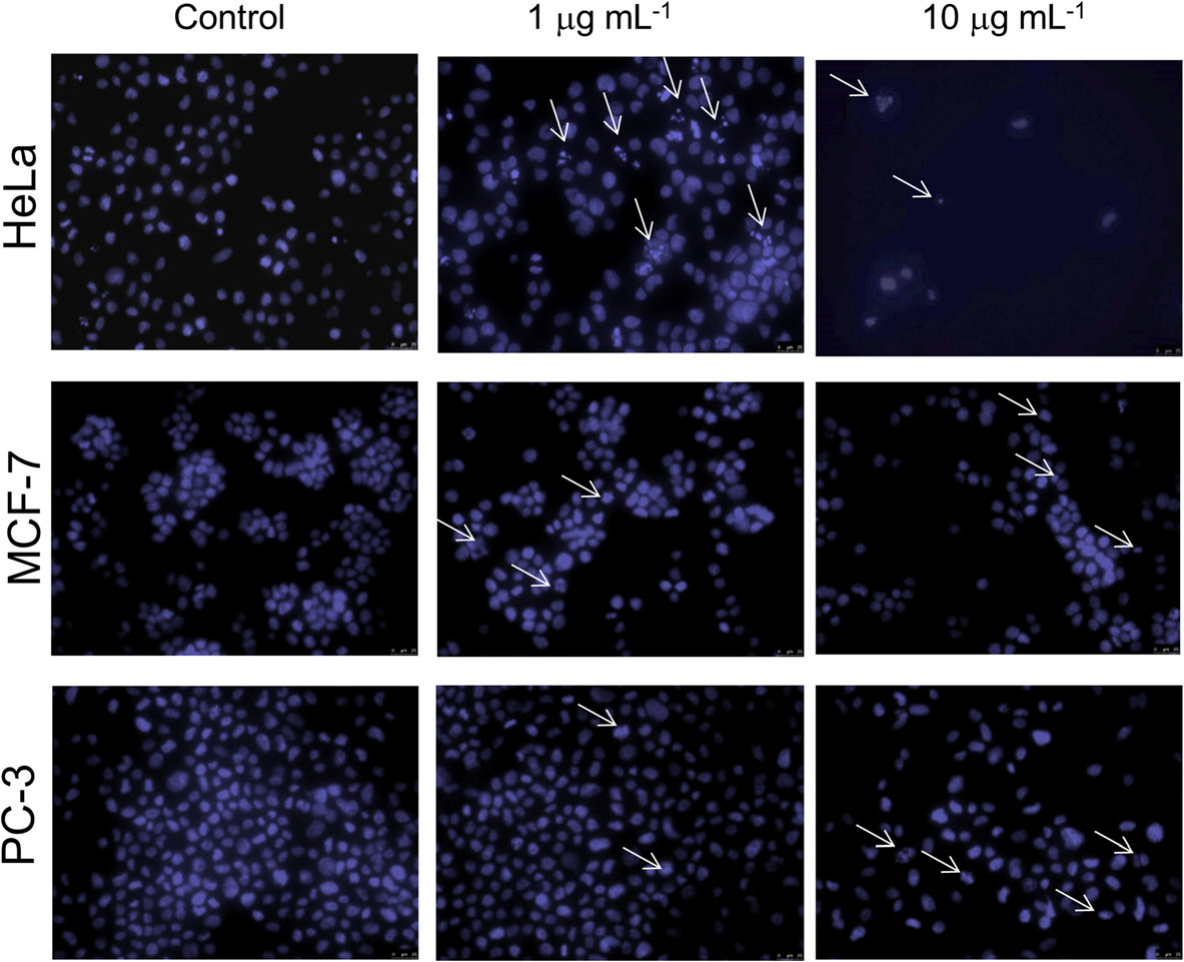
Morphology of HeLa, MCF-7 and PC-3 cancer cells treated with different concentrations of the extract. ^a^Cells were treated with the extract from mycelium of *Caloplaca pusilla* grown on G-LBM medium at different concentrations (0, 1, or 10 μg mL^−1^) for 48 h. Cells were stained with DAPI and examined under a fluorescence microscope. Examples of apoptotic cells and/or bodies are indicated by arrows

We confirmed induction of apoptosis in all tested cell lines treated with 1 μg mL^−1^ or 10 μg mL^−1^ concentration of the extract from mycelium of *Caloplaca pusilla* grown on G-LBM medium by DAPI staining of cell chromatin. Cells undergoing apoptosis revealed highly condensed chromatin which at later stages became fragmented and packed into apoptotic bodies detected in samples treated with the analyzed extract. Moreover, higher concentrations of the extract caused cell loss which might result from a secondary necrosis (Fig. 4).

## Discussion

In the present study the antibacterial and cytotoxic activities of mycelial extracts from in vitro cultured lichen-forming fungi were tested. We isolated and successfully cultured *Caloplaca pusilla, Protoparmeliopsis muralis* (syn. *Lecanora muralis*) and *Xanthoria parietina.* Previous studies by Ranković and co-workers [25–27] reported that acetone extracts from lichen *Protoparmeliopsis muralis* inhibited the growth of selected bacteria (MIC ranged from 1.56 to 6.25 mg mL^−1^) and showed antifungal activity against e.g. *Botrytis cinerea* and *Paecilomyces variotii*. Here we show that aposymbiotically cultured lichen-forming fungus *P. muralis* can be a source of antibacterial substances, therefore it limits the need to harvest lichen thalli in the field. In this study *P. muralis* showed the strong inhibitory effects on the growth of Gram-positive bacteria, i.e. *Bacillus subtilis*, *Enterococcus faecalis, Staphylococcus aureus* and *Staphylococcus epidermidis* (MICs from 6.67 to 100.00 μg mL^−1^ depending on the bacterial strain and type of extract) (see Tables 1 and 2). However, it is very important to choose an appropriate medium for lichen-forming fungi culture, as extracts from mycelia grown on PDA medium did not show any antibacterial activities while culturing on G-LBM, G-LBM+ and MY media resulted in the production of antibacterial substances present in acetone extracts from mycelia.

Because of its color, *Xanthoria parietina* has been used against jaundice in traditional medicine since antiquity. In eastern Andalucia (Spain) *X.* parietina was used to treat menstrual complaints, kidney disorders and as an analgesic [28]. Extracts from *Xanthoria parietina* were tested against several bacterial strains and it was found that acetone extracts from *X. parietina* inhibited all the tested microorganisms at concentrations of 7.8–62.5 μg mL^−1^. The main secondary metabolite of this lichen, i.e. parietin, displayed a robust antibacterial activity, with MICs ranging from 7.8 to 62.5 μg mL^−1^ [29]. However, in our study extracts of in vitro cultured mycelia of *Xanthoria parietina* did not show any antibacterial activity against tested bacteria, at least at the concentrations used by us (Table 1).

Moreover, acetone extracts from *Xanthoria parietina* showed a dose-dependent antiproliferative activity against MCF-7 and MDA-MB231 cells, with the greatest effect obtained at the concentration of 1.5–3 mg mL^−1^ of the extract [29]. In our study in vitro cultured mycelia of *Xanthoria parietina* grown on PDA (labelled as Xp_PDA) and G-LBM (labelled as Xp_GLBM) media proved to be a source of anticancer substances. They showed strong potency in decreasing the cancer cells viability with IC_50_ values of about 8 μg mL^−1^ for HeLa or MCF-7 cell lines (Table 3).

In the previous studies the extracts from *Protoparmeliopsis muralis* (syn. *Lecanora muralis*) showed a cytotoxic activity against FemX, LS174 and HCT-116 cell lines with IC_50_ of 9.58, 12.23 and 331.11 μg mL^−1^, respectively [25, 27]. In our study we did not detect any antiproliferative effect of mycelial extracts from *Protoparmeliopsis muralis* against HeLa, MCF-7 nor PC-3 cell lines at the tested concentrations. However, the other nutrient media used for mycelial growth might stimulate the synthesis of other compounds with anticancer properties.

So far, there were no data on antibacterial or antiproliferative activity of *Caloplaca pusilla*. In our study we did not detect any antimicrobial activity of its acetone extracts. However, *Caloplaca pusilla* grown on G-LBM medium (Cp_GLBM) showed the highest potency in decreasing the cancer cell viability with IC_50_ values of 6.57 μg mL^−1^ for HeLa cell line, 7.29 μg mL^−1^ for MCF-7 and 7.96 for PC-3 cell lines. The effect was dose-dependent and connected with induction of apoptosis.

In this study we showed that the growth of mycelium is dependent on the medium used. For each mycobiont the optimal medium should be selected experimentally as lichen-forming fungi show different ecological preferences. In case of *Protoparmeliopsis muralis* and *Xanthoria parietina* all media were appropriate for their growth, while the growth of *Caloplaca pusilla* was much better on MY or PDA medium in comparison to G-LBM and G-LBM+ media.

Moreover, we showed that the biological activity of mycelial extract differs depending on culture conditions of the lichen-forming fungus. In case of *P. muralis*, mycelia grown on MY, G-LBM or G-LBM+ media showed strong antibacterial effect, while the same fungus grown on PDA medium did not show any activity. Extract from the mycelium grown on G-LBM+ was the most active and showed better antimicrobial activity than the extract from the thallus of *P. muralis* which suggests that in vitro cultured mycelia may synthesize other metabolites and/or higher quantity of antimicrobial compounds. In case of other lichens we did not observe any antimicrobial effect.

In this study we also showed antiproliferative activity of acetone extracts from *Caloplaca pusilla* and *Xanthoria parietina*. Extract from *Protoparmeliopsis muralis* did not show antiproliferative effect although previous studies reported that compounds extracted from *P. muralis* inhibit the growth of selected cancer cell lines [25–27]*.* Although the growth of *C. pusilla* on G-LBM was limited, its extract showed the highest potency in decreasing the cancer cells viability, while the same mycobiont grown od PDA did not show any antiproliferative effect.

Although lichens have been used in folk medicines for ages (for review see Crawford [5]) their antibiotic, antiproliferative, antimutagenic, antifungal properties are continuously studied [5, 6, 27, 29–32]. As lichens can be a potential source of pharmaceutically useful chemicals [7–11] one should bear in mind difficulties in sourcing lichen substances required for various applications. Lichens are relatively slow-growing organisms and their intensive harvesting is not ecologically acceptable [12]. Importantly, they can be grown in in vitro cultures [13–15]. However, culture conditions should be tested and well selected for each species individually [15]. Moreover, culture conditions and nutrient media may change the quality and quantity of the metabolites produced by mycobiont. It gives us the opportunity to obtain novel or highly concentrated substances with interesting properties. However, our knowledge about the substances secreted by separated bionts is scarce and needs to be investigated.

## Conclusions

In this study we demonstrated that aposymbiotically cultured lichen-forming fungi might be a source of antibacterial and antiproliferative substances/compounds. Optimization of culture conditions may accelerate their growth and stimulate the production of biologically active compounds. It may also limit the need to harvest lichen thalli in the field. It is especially important for lichens with small thalli such as *Caloplaca pusilla*. A high variety of biologically active substances present in mycelial extracts may provide new antibacterial and/or anticancer agents.

## Abbreviations

Cp: *Caloplaca pusilla*
DMSO: Dimethyl sulfoxide
G-LBM: modified LBM medum
MBC: minimal bactericidal concentration
MIC: Minimal inhibitory concentration
MY: malt extract-yeast extract medium
PDA: Potato-Dextrose Agar
Pm: *Protoparmeliopsis muralis*
Xp: *Xanthoria parietina*
